# Vitamin E Attenuates Red-Light-Mediated Vasodilation: The Benefits of a Mild Oxidative Stress

**DOI:** 10.3390/antiox13060668

**Published:** 2024-05-29

**Authors:** Agnes Keszler, Dorothee Weihrauch, Brian Lindemer, Grant Broeckel, Nicole L. Lohr

**Affiliations:** 1 Department of Medicine, Division of Cardiovascular Medicine, Medical College of Wisconsin, Milwaukee, WI 53226, USA; akeszler@mcw.edu (A.K.); dorothee@mcw.edu (D.W.); blindeme@mcw.edu (B.L.); gbroeckel@mcw.edu (G.B.); 2 Department of Plastic Surgery, Medical College of Wisconsin, Milwaukee, WI 53226, USA; 3 Cardiovascular Center, Medical College of Wisconsin, Milwaukee, WI 53226, USA; 4 Clement J. Zablocki VA Medical Center, Milwaukee, WI 53295, USA; 5 Department of Medicine, Division of Cardiovascular Disease, University of Alabama at Birmingham, Birmingham, AL 53233, USA; 6 Birmigham VA Medical Center, Birmingham, AL 53233, USA

**Keywords:** photobiomodulation, red-light therapy, vasodilation, oxidative stress, exocytosis

## Abstract

Red light (670 nm) energy controls vasodilation via the formation of a transferable endothelium-derived nitric oxide (NO)-precursor-containing substance, its intracellular traffic, and exocytosis. Here we investigated the underlying mechanistic effect of oxidative stress on light-mediated vasodilation by using pressure myography on dissected murine arteries and immunofluorescence on endothelial cells. Treatment with antioxidants Trolox and catalase decreased vessel dilation. In the presence of catalase, a lower number of exosomes were detected in the vessel bath. Light exposure resulted in increased cellular free radical levels. Mitochondrial reactive oxygen species were also more abundant but did not alter cellular ATP production. Red light enhanced the co-localization of late exosome marker CD63 and cellular S-nitrosoprotein to a greater extent than high glucose, suggesting that a mild oxidative stress favors the localization of NO precursor in late exosomes. Exocytosis regulating protein Rab11 was more abundant after irradiation. Our findings conclude that red-light-induced gentle oxidative stress facilitates the dilation of blood vessels, most likely through empowering the traffic of vasodilatory substances. Application of antioxidants disfavors this mechanism.

## 1. Introduction

Initial observations of light energy on tissues were first described in the 1960s when application of a ruby laser improved wound healing [1,2]. Photobiomodulation (PBM), the therapeutic application of energy in the red and near infrared (NIR) spectrum, is gaining widespread adoption. Energy sources can be a laser or a light emitting diode (LED), with variable wavelengths (630–1000 nm) and energy levels pre-determined by the investigated biological activities and therapeutic usefulness [3]. The variety of experimental studies and treatments applying photobiomodulation, such as wound healing, myocardial infarction, traumatic brain injury, endothelial disfunction, etc., Refs. [4,5], renders diversity in its underlying mechanisms. The primary targets of the light are various cellular photo acceptor molecules, which upon stimulation, trigger secondary effects, e. g., expression or stimulation of second messengers, modulation of gene expressions, and enzyme activities [5,6]. The canonical mechanisms involve the light absorption in the mitochondria, with subsequent release of critical vasodilatory species nitric oxide (NO) from complex IV of the electron transport chain, generation of reactive oxygen species (ROS), and enhanced ATP production [4,6]. 

More recent studies offered alternative ways of NO formation by release from derivatives outside the mitochondria using energy of the far-red region of the electromagnetic spectrum [7,8] or by phosphorylation of the eNOS enzyme upon light exposure in the infrared range [9]. However, there is a higher consensus on the mitochondrial relation of the light-controlled ROS release [10,11,12], although general cellular ROS formation via a possible NADPH oxidase-dependent mechanism was also reported [12]. Near infrared light induces more ROS than far red light, and a structured intracellular water layer is also involved in the mechanism at high wavelengths [10]. Alternatively, PBM, besides generating oxidative stress on its own, has been shown to reduce severe oxidative damage that occurs under pathological conditions [13] or post exercise [14] by improving the activity of innate antioxidants and decreasing ROS biomarker levels.

Our previous research focused on the critical role of red light in facilitating vasodilation [15,16,17]. We established that 670 nm energy is optimal [7], and suggested a mechanism for the formation, intracellular traffic, and exocytosis of endothelium-derived, transferable, NO-precursor-containing vesicles [16,17]. In this study, we hypothesized that red light controls vital elements of vasodilatory vesicle trafficking through ROS generation. We investigated the contribution of the red-light-generated ROS to vasodilation by using vitamin E derivative Trolox and catalase. Moreover, we studied the 670 nm light-mediated changes in cellular free radicals and mitochondrial ROS and compared the red light exerted impact with that of hyperglycemia, a potent inducer of oxidative stress. Lastly, we examined the effect of oxidative stress on the localization of NO precursors in the late exosomes, and on expression of exocytosis-regulating proteins. We found that the 670 nm energy-stimulated oxidative stress is mild and increases the dilation of facialis arteries dissected from C67BI/6 mice, presumably via supporting the traffic of vasodilatory substances.

## 2. Materials and Methods

### 2.1. Materials

Trolox was purchased from Cayman Chemical Co. (Ann Arbor, MI, USA); anti S-nitrosocysteine (SNO-Cys) was a Creative Diagnostics (Shirley, NY, USA) product; anti Rab11, anti Rab5, DMPO, and anti DMPO were obtained from Abcam (Waltham, MA, USA). Cell culture medium, anti CD63, and DAPI stains came from ThermoFisher Sci. (Waltham, MA, USA). Alexa 488 and 610 conjugated secondary antibodies were from Santa Cruz Biotech (Dallas, TX, USA). Fetal Bovine Serum (FBS) was bought from ATCC (Manassas, VA, USA) and mounting medium for immunofluorescence was from Triangle Biomedical Sci. (Durham, NC, USA). Other chemicals were procured from Sigma-Aldrich Co. (St. Louis, MA, USA). LED light sources and power supply were made by Quantum Devices Inc. (Barneveld, WI, USA); the output was measured with an X97 Optometer (Gigaherz Optic Gbmh, Turkenfeld, Germany).

### 2.2. Pressure Myography

C57BL/6 mice (25.2 ± 0.3 g) were purchased from Jackson Laboratories. All procedures and protocols used conformed to the Guiding Principles in the Care and Use of Animals of the American Physiologic Society, were in accordance with the Guide for the Care and Use of Laboratory Animals and reviewed and approved by the Animal Care and Use Committtee of the Medical College of Wisconsin. Facialis arteries segments (160–260 µm) were dissected and transferred to a water-jacketed perfusion chamber and cannulated with two glass micropipettes (ID 30 µm). They were bathed in physiological saline solution (PSS)-equilibrated solution and maintained at pH 7.4 at 37 °C. The micropipettes were connected; PSS and vessels were pressurized at 60 mmHg. Internal diameter was determined with a stereomicroscope (Olympos CK 40, Olympos America Inc. Melville, NY, USA), a charge-coupled device camera (Panasonic GP-MF 602, Matsuda Electric Corporation of America, Secaucus, NY, USA), and a video measuring apparatus (Boeckeler VIA-100, Boeckeler Instruments, Inc. Tucson, AZ, USA). After 1 h the arteries were pre-constricted by 50% with U-46619. The vessels were kept in dark until steady-state contraction was achieved then irradiated (670 nm, 10 mW/cm^2^, 10 min). Control vessels were kept in dark for 10 min.

### 2.3. Exosome Tracking

Exosomes were separated from the vessel bath with centrifugation at 2000× *g* for 10 min at 4 °C followed by ultracentrifugation of the supernatant at 55,000× *g* for 1 h at 4 °C. The pellet was resuspended in 500 µL of phosphate buffer saline (PBS). Exosomes were counted by Nanoparticle Tracking Analysis (NanoSight NS 300, Malvern Instruments Ltd. Malvern, UK).

### 2.4. Cell Culture

Bovine Aortic Endothelial Cells (BAECs) were obtained from Cell Applications Inc. (San Diego, CA, USA) and cultured under standard conditions using media containing RPMI, 20% FBS, 2 mM L-glutamine, and 0.5% Penicillin/Streptomicine. All experiments were performed using passages P5–P9. Cells were plated on 100 mm TPP (trademark of TTP Techno Plastic Products Products AG) cell culture dishes (Midwest Scientific, Valley Park, MO, USA) or four chamber cell culture slides (CELLTREAT Scientific Products, Pepperell, MA, USA) pre-treated with 2% gelatin prior to seeding.

### 2.5. Immunofluorescence

BAECs were cultured on slides. Slides were post fixed with 4% paraformaldehyde, and cells were permeabilized in 0.5% Triton X for 5 min at room temperature. Samples were incubated with the primary antibody (30 min at 37 °C), and after washing with PBS, they were treated with Alexa 488 or 610 secondary antibodies (30 min at 37 °C). Alternatively, to detect mitochondrial ROS MitoPy, dye was applied for 5 min at room temperature. DAPI was used for nuclear staining (for 5 min at room temperature). Slides were mounted with Shur/Mount medium, and cover slipped. Images were captured and quantified with a Nikon Eclipse Ti2 fluorescent microscope equipped with NIKON Element (5.02.00) software. Pixel intensities were determined after background compensation. The threshold was maintained the same through experimental repetition to minimize variability.

### 2.6. Adenine Nucleotide Analysis

Cellular adenine nucleotides (ATP, ADP, AMP) were separated and quantified using the HPLC method with spectrophotometric detection at 262 nm, based on published methods [18,19]. Briefly, after aspirating the medium, cells were lysed in 5% ice-cold perchloric acid, then neutralized with 1 M K_2_HPO_4_. The suspension was kept on ice for 10 min, then centrifuged at 10,000× *g* for 5 min at 4 °C. Protein content was determined from the pellets suspended in 0.5 N NaOH. The supernatant was diluted with an equal amount of Solvent A and filtered through a 0.2 µm Corning filter. The separation was performed on a 250 mm (4.6 mm ID, 0.5 µm partcle size) C-18 Kromasil column (Supelco Inc., Bellefonte, PA, USA) using an Agilent 1100 chromatograph. The mobile phase consisted of 0.1 M potassium phosphate, 4 mM tetrabutylammonium bisulfate, pH 6.0 diluted 64:36 (*w*/*w*) in dd water for Solvent A, or in methanol for Solvent B. The gradient method was applied starting with 100% of Solvent A followed by increasing Solvent B concentration to 30% in 2.5 min, then to 50% in 5 min. It was further increased to 65% in 10 min and maintained for 5 min, then decreased to 0% in 4 min. Solvent A was kept at 100% for 11 min. Peaks were integrated with Agilent ChemStation (B. 03.01.) software and quantified by known amounts of nucleotides. 

### 2.7. Statistics

**Figure 1:**
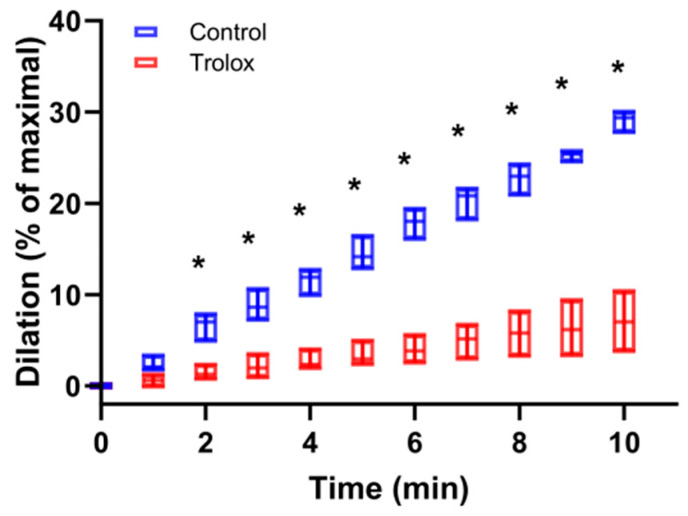
Antioxidant Trolox attenuates ex vivo vasodilation. Facialis arteries from C67BI/6 mice were isolated and pressurized before constriction. Red light (670 nm, 6 J/cm^2^) was applied in the absence and presence of Vitamin E derivative Trolox (200 µM). Dilation was assessed with pressure myography and expressed as a percentage of maximal dilation. The Trolox-dependent decrease was significant from t = 2 min with * *p* < 0.05 by using a paired Student’s *t* test. N = 6.

**Figure 2:**
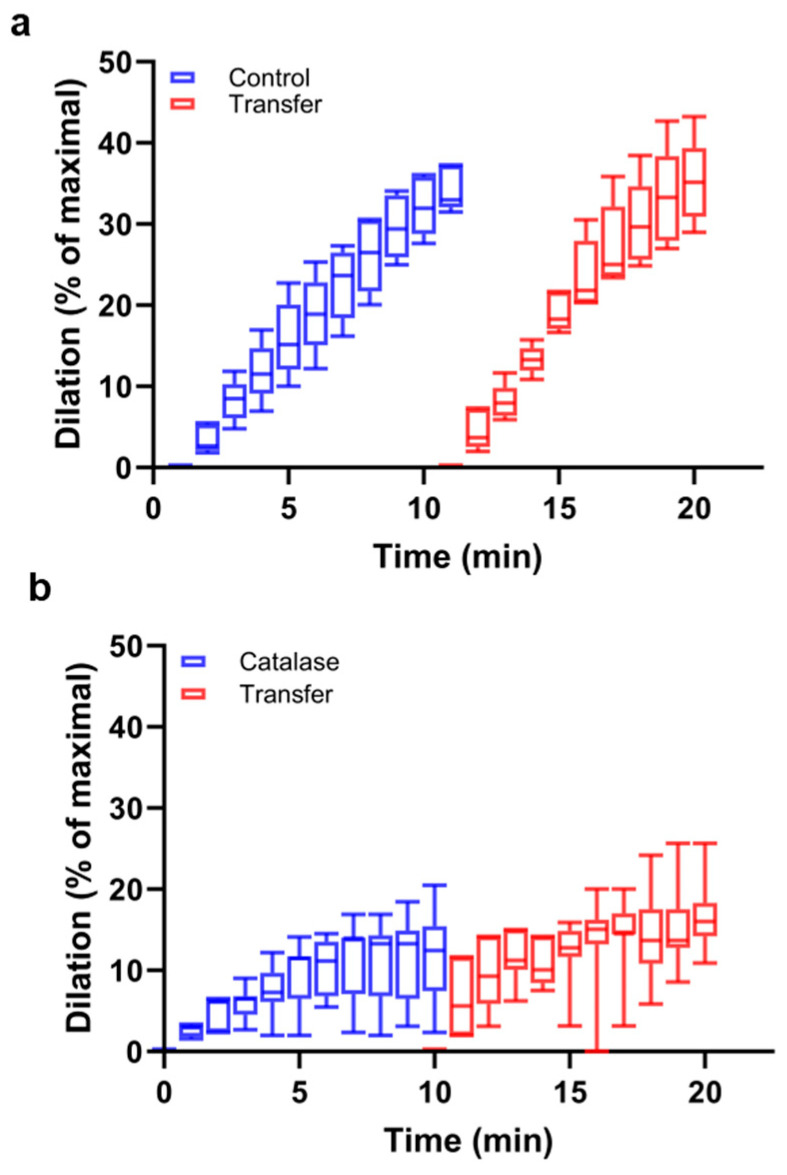

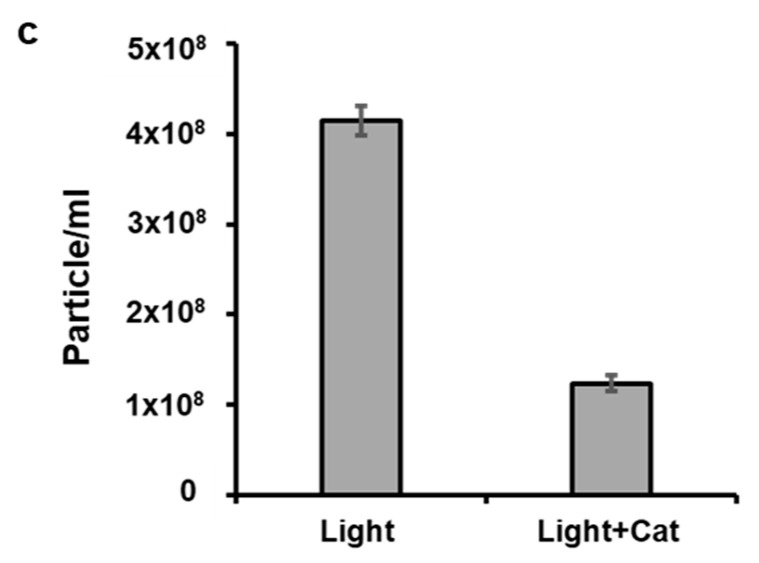
Adoptive transfer with catalase. Facialis arteries from C67BI/6 mice were isolated and pressurized before constriction. (**a**): Red light (670 nm, 6 J/cm^2^) induced vasodilation, and the bath caused equivalent dilation of the naïve vessel (*p* > 0.05 at all the time points). N = 5; (**b**): catalase (100 U/mL) pre-treatment significantly decreased the light-mediated vasodilation (*p* < 0.05, by using a paired Student’s *t* test, from 2 min through 10 min compared to non-catalase). This bath transferred to the naïve vessel resulted in a similar decreased dilation. N = 7; (**c**): catalase decreased the exosome concentration in the vessel bath measured with Nanoparticle Tracing Analysis. The Y axis shows the overall concentration of all detected particles of various sizes. The particle distribution varies between 35 and 700 nm. Bars represent means ± SD. N = 5.

One-tailed Student’s *t* test (paired for Figure 1 and Figure 2a,b, unpaired in all other cases) was used for statistical analysis, and a *p* < 0.05 value was considered statistically significant. Data are reported as bars and whiskers set to 5–95% unless otherwise indicated. All graphs were prepared using GraphPad Prism 10 software, except Figure 2c, which was made in Microsoft Excel.

## 3. Results

### 3.1. Effect of Trolox and Catalase on Red-Light-Mediated Dilation of Ex Vivo Blood Vessels

#### 3.1.1. Trolox

Previous studies indicate the application of 670 nm energy enhances the dilation of a dissected murine facialis artery in a NO-related manner [16]. Here we examine the effect of ROS on the red-light-dependent dilation by treating vessels with antioxidants. One obvious agent would be ascorbate or vitamin C, the most abundant water-soluble antioxidant in the circulation [20]; however, ascorbate also reacts with a major active vasodilatory species, S-nitrosothiol (RSNO), and loses its vasoactivity via reduction to a thiol compound [21,22]. Therefore, we chose Trolox, a water-soluble vitamin E derivative which is one of the most efficient water-soluble antioxidants against strong oxidant hydroxyl radicals [23] which does not interfere with RSNO [24]. 

When applying 200 µM Trolox dissolved in the bath buffer of preconstricted facialis artery obtained from C67Bl/6 mice, the 670 nm light-induced dilation decreased in 10 min from 28.0% to 7.1% of the maximal value (Figure 1). The difference was significant at every time point starting from 2 min and suggested that oxidative stress is a positive contributor to light-mediated dilation of blood vessels.

#### 3.1.2. Catalase

Further, we tested whether the antioxidant enzyme catalase, which protects cells from oxidative stress by converting H_2_O_2_, the precursor of hydroxyl radical, to H_2_O would act similarly to Trolox. Indeed, catalase pretreatment (100 U/mL) resulted in a reduction of dilation from 34.3% (Figure 2a, blue) to 12.0% (Figure 2b, blue) in 10 min. Again, the difference was significant at all measured time points starting from 2 min. To test whether the antioxidant treatment would interefere with the production of a transferrable vasodilator, the bath buffer, after light exposure of the primary blood vessel, was transferred to a naïve artery in the presence and absence of catalase. As expected, the attenuating effect of catalase was transmittable to a naïve vessel, indicating the dilating effect was not weakend during transfer to the bath (Figure 2a, red and Figure 2b, red). This is consistent with our earlier findings [16] and suggests the formation and traffic of extracellular vesicles could be responsible for the observed vasodilation [17] and are regulated by the light-dependent generation of ROS. To test this hypothesis, the bath buffer after light treatment was collected and subsequently underwent particle testing with Nanoparticle Tracking Analysis after catalase pre-treatment (1.24 × 10^8^ ± 8.23 × 10^6^ particle/mL) as compared to the untreated control (4.15 × 10^8^ ± 1.6 ×10^7^ particle/mL). These results support a decrease in exosome concentration in the presence of catalase. The automated measurements provide the results as mean ± SD and do not compute significance, but the 3.3-fold drop with the tight error intervals suggest a remarkable difference (Figure 2c). 

### 3.2. Effect of 670 nm Light on Reactive Oxygen Species (ROS) Levels

#### 3.2.1. Microsomal and Cellular Free Radicals 

The significant attenuation of antioxidants on blood vessel diameter required further investigation of released ROS as a mechanism for light-mediated vasodilation. Reactive oxygen species initiate oxidation of cellular lipids and proteins on the hydrocarbon sidechain [25,26] and are early markers of oxidation. ROS can be detected by using the spin trap DMPO, which upon reaction with ephemeral radicals eventually produces a stable nitrone compound. When the primary radicals are formed on a protein or DNA residue, a DMPO-specific antibody can be used to recognize the end-product [27]. Thus, we applied an immunofluorescence technique to visualize free radicals in cultured endothelial cells (BAECs) challenged with 670 nm light using the anti-DMPO antibody.

**Figure 3:**
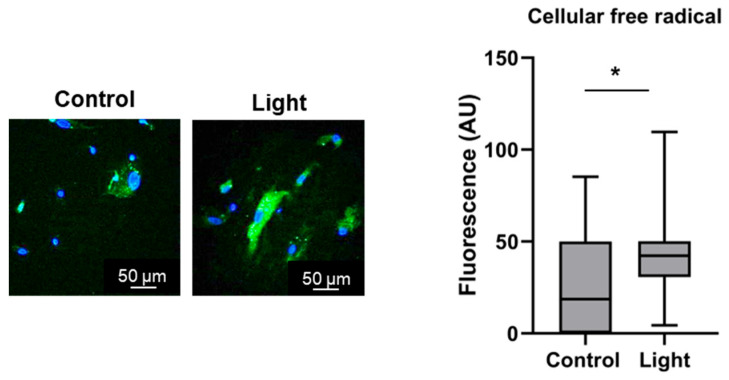
Red-light-mediated free radical formation. Free radicals were recognized with an anti-DMPO antibody. Endothelial cells (BAECs) were incubated with DMPO (100 mM, 1 h), then exposed to light (670 nm, 6 J/cm^2^). Free radicals were detected using an anti-DMPO antibody (green) and nuclei were stained with DAPI (blue). A significant fluorescence increase of intensity was detected after light exposure (* *p* < 0.05 by using a one-tailed unpaired Student’s *t* test). N = 15 slides.

Irradiation of DMPO pre-treated BAECs resulted in a significant 50% increase of anti-DMPO fluorescence compared to the dark control (Figure 3). These results indicate that 670 nm light energy triggers the formation of free radicals in the endothelium. 

#### 3.2.2. Mitochondrial ROS

The majority of ROS are formed in the mitochondria [28], whose antioxidant system controls the dynamics of ROS metabolism, particularly the H_2_O_2_ balance [29]. It has been widely accepted that one action of PBM is to modulate ROS levels and ATP production through targeting the complexes of the electron transport chain in a wavelength dependent manner [4,5,10]. Typically, application of a longer wavelength results in more increased stress, and the fluence has smaller effect, while the ATP response is more sensitive to both wavelength and fluence [10].

**Figure 4:**
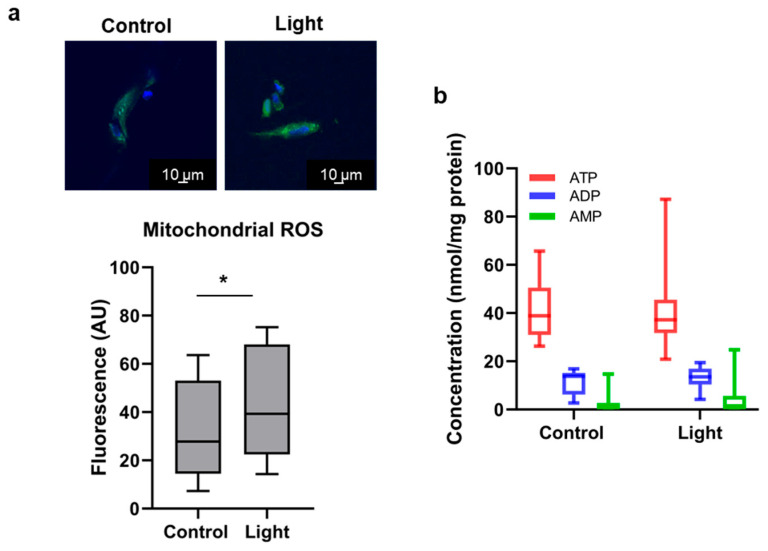
Effect of red-light mediated oxidative stress on mitochondria. BAECs were exposed to light (670 nm, 6 J/cm^2^) (**a**) Mitochondrial ROS was detected with MitoPy fluorescence (green). Light exposure resulted in significant increase (* *p* < 0.05). Nuclei were stained with DAPI (blue). N = 9 slides; (**b**) Changes in levels and distribution of adenine nucleotides were determined in cell lysate after HPLC separation. Between control and light, *p* > 0.05 and N = 13 for all ATP, ADP, and AMP. A one-tailed unpaired Student’s *t* test was used.

Using MitoPy, a mitochondrion specific fluorescent marker of H_2_O_2,_ we detected a significant 34.7% increase in fluorescence intensity after challenging the cells with 670 nm light (Figure 4a). However, when measuring the ATP concentrations in the cell lysate after separation on HPLC, the values fluctuated around 41 nmol/mg protein without a significant difference between irradiated and dark control samples (Figure 4b). Likewise, the levels and distribution of other adenine nucleotides (ADP and AMP) have not changed either. These observations suggest that the conditions found optimal to increase vasodilation also stimulate a mild oxidative stress with maintained ATP production.

### 3.3. Effect of Red-Light-Induced Oxidative Stress on Exosome Formation and Release

We have evidences [16,17] that the red-light-mediated vasodilation occurs via the release of exosomes containing dilatory species from the endothelium and presumably with a subsequent transfer to the smooth muscle. It is well described that oxidative stress can increase exosome yield and regulate its cargo in diverse cell types as a response to various stimuli [30,31]. 

Therefore, we investigated how different degrees of oxidative stress modulate the formation and content of exosomes. High glucose concentrations increase ROS levels through cytosolic oxidation, resulting in an elevated NADH concentration and subsequent superoxide production in the mitochondria [32]. Therefore, we compared, via the expression of late exosome marker protein CD63, how high glucose and red light can modulate exosome concentration and whether the vasodilatory cargo is affected.

**Figure 5:**
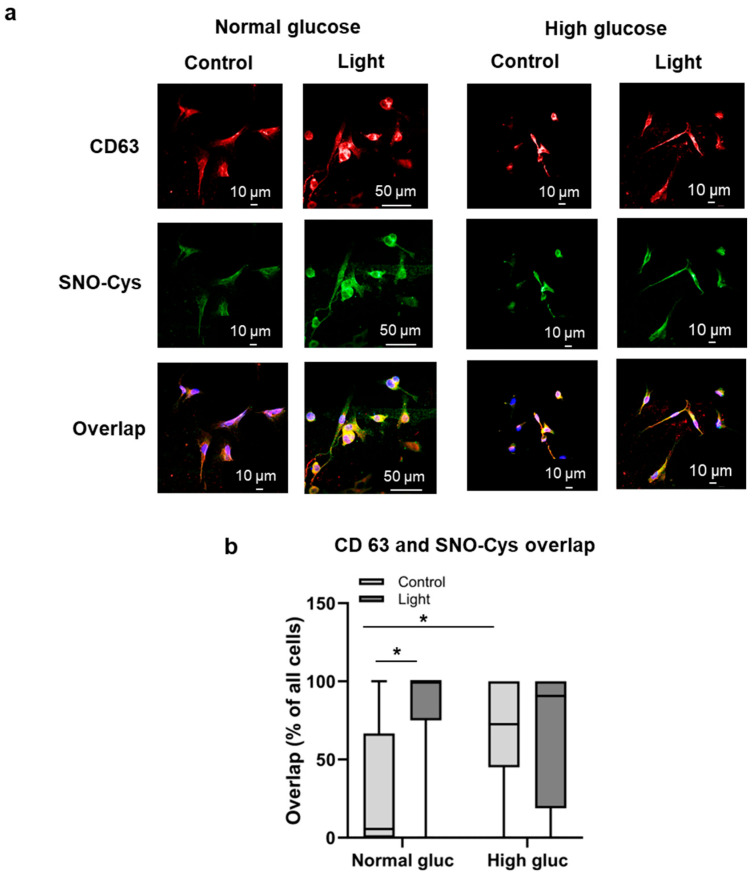
Effect of red light and glucose on the localization of vasodilatory substance. BAECs were pre-incubated with D-glucose (15 mM, overnight) and exposed to light (670 nm, 6 J/cm^2^). Co-localization of CD63 late exosome marker with S-nitrosoproteins (SNO-Cys) characterizes the accumulation of vasodilatory SNO-Cys in the late exosomes. (**a**) Representative images. CD63 is labeled as red and SNO-Cys as green. Nuclei were stained with DAPI (blue); (**b**) quantification of the overlap between CD63 and SNO-Cys (* *p* < 0.05 by using a one-tailed unpaired Student’s *t* test). N = 6 slides.

Glucose concentration was reported to promote exosome discharge from cells as measured by the expression of CD63 [33], which is consistent with our findings; however, red light exposure did not provide a further increase, regardless of the glucose concentration (Figure S1). On the other hand, irradiation enhanced the presence of key vasodilatory compound RSNO in the vesicles. We pre-incubated BAECs with D-glucose (15 mM, overnight), double-labeled them with antibodies against CD63 and S-nitrosocysteine (SNO-Cys), and examined their co-localization (Figure 5). Co-localization is displayed as yellow, resulting from overlapping CD63 (red) and SNO-Cys (green) and was quantified by the number of cells containing yellow color as a percentage of all cell numbers. The results revealed an abundant light-dependent increase of overlap between the two proteins (82.0% after irradiation vs 30.6% in the dark control) in the presence of normal glucose concentrations. Likewise, higher glucose content resulted in a greater overlap in the non-irradiated samples (63.3% vs. 30.6% with normal glucose). Importantly, the 670 nm energy elicited a significantly stronger effect than the elevated glucose concentration. When the glucose pre-treated cells were irradiated, no further growth of overlap was observed. The results indicate that both the red light and the high glucose derived stress results in enhanced RSNO enrichment in the exosomes, but the effect of red light is more pronounced. These findings suggest that application of red light is not just harmless, unlike high glucose, it is also a more advantageous regulator of RSNO traffic.

The elevated free radical levels detected in the cells and the higher mitochondrial ROS imply the participation of reactive oxygen species in the red-light-derived release of vasodilatory vesicles. To monitor this effect, we compared how 670 nm light and H_2_O_2_ beyond the homeostatic concentration [34] would influence the expression of peripheral membrane protein Rab11, which is located in the secretory vesicles and has a decisive role in controlling exocytosis [35,36]. 

**Figure 6:**
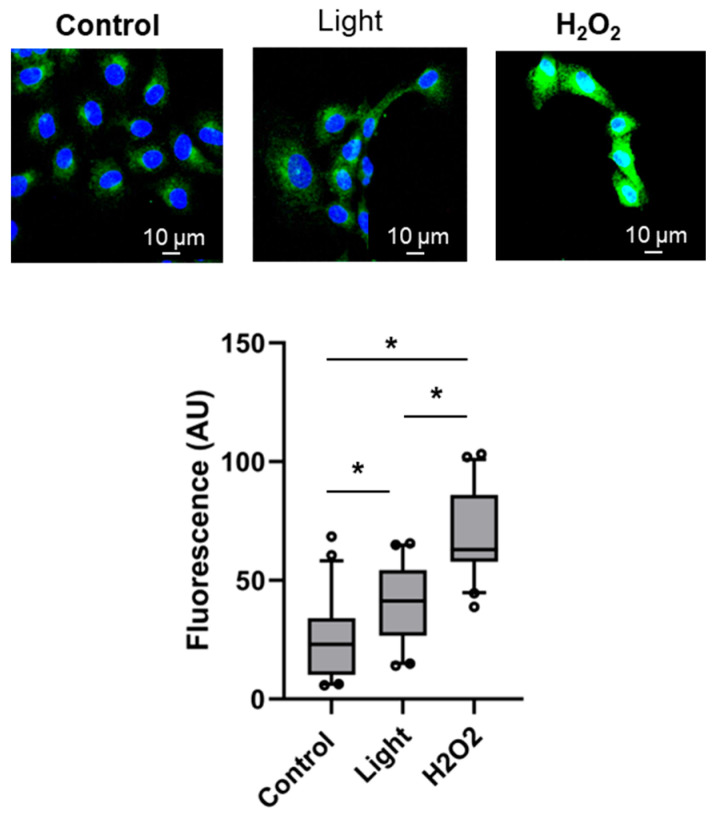
Comparison between the effects of mild (red light) and strong (H_2_O_2_) oxidative stress on exocytosis-regulating membrane protein Rab11. Endothelial cells were exposed to light (670 nm, 6 J/cm^2^), or treated with H_2_O_2_ (100 µM, 1 h). Rab11 levels were detected with immunofluorescence using the corresponding antibodies (green). Significantly higher intensities were recorded with H_2_O_2_ vs. light exposure (* *p* < 0.05 by using a one-tailed unpaired Student’s *t* test). Nuclei were stained with DAPI (blue). N = 12 slides.

The red light exposure significantly enhanced the Rab11 levels by 58.8% compared to the dark control, as measured with immunofluorescence, while H_2_O_2_ pre-treatment (100 µM, 1 h) resulted in a much larger 171.8% increase (Figure 6), indicating the mild stress that 670 nm energy elicits is enough to induce exosome release. Interestingly, Rab5, a characteristic protein of the early endosomes [37], was not sensitive to irradiation, although its presence was more abundant after H_2_O_2_ pre-treatment (Figure S2), demonstrating red light energy rather contributes to the intercellular traffic of cargo than early intake through endocytosis.

## 4. Discussion

This study is focused on characterizing the underlying contribution of ROS production to the complex mechanism of red-light-dependent vasodilation.

Our laboratory investigates the exploitation of non-enzymatic internal NO stores by using red and NIR light to induce dilation of ex vivo blood vessels [7,16,17] and elaborated an in vivo model system to mimic peripheral artery disease and alleviate its symptoms [15]. The observed physiological effects from these light sources are independent of temperature increases. Mechanistically, we have established that 670 nm light delivers the optimal energy to mobilize endothelial NO precursors consisting of mostly RSNO. The active vasodilator is embedded in vesicles, therefore sheltered from degradation, and is chemically and physiologically stable for at least 30 min after exiting the endothelium [15,17]. The applied red light controls the formation of the vesicles, their intracellular transfer, and the exocytosis [38]. 

Extracellular vesicles are cytosolic particles of various sizes with versatile cargo, surrounded by a lipid bilayer [39]. Multiple pathways and cellular organelles are involved in their generation and traffic through the endosomal system from early endosome formation to exosome secretion via the late endosomes or multivesicular bodies (MVB) [40], and ROS may serve as stimulator in their journey [41]. Here, we hypothesized that 670 nm energy regulates critical steps in the trafficking of vasodilatory vesicles through ROS generation. We found that (i) Trolox and catalase decreased the red-light-induced dilation of pre-constricted facialis arteries of mice, and the vessel bath buffer carried over a decreased number of dilatory vesicles compared to control (Figure 1 and Figure 2); (ii) irradiation increased the levels of cellular free radicals and mitochondrial ROS (Figure 2 and Figure 3); (iii) light exposure boosted the localization of vasodilatory species in the vesicles (Figure 5) and enhanced the abundance of exocytosis-regulating protein Rab11 (Figure 6). According to published data, numerous red and NIR light-stimulated cellular activities, e.g., proliferation and viability, can be reversed by antioxidant supplementation [42], which is in line with the observed effect of Trolox and catalase on ex vivo vessel dilation. The abundance of free radicals is another marker of an oxidative environment, which facilitated the expression of CD63, a protein that is predominantly enriched on the exosome surface [39]. The oxidative milieu is also favorable for exosome secretion, as is shown by the amplified presence of Rab11, which actively participates both in the formation of recycling vesicles and in their exocytosis at the plasma membrane [35]. 

Remarkably, the cellular expression of Rab11 increased to a lesser extent after light exposure than after a pre-treatment with a stronger oxidant H_2_O_2_ (Figure 6). On the other hand, the mild energy of 670 nm light triggered a more pronounced vesicular localization of vasodilatory RSNO than high glucose stress (Figure 5). This raises the question of what role the different strengths of ROS may play in PBM therapies. The ROS generation is dependent on wavelength, dose, and time. A biphasic response to oxidative stress was reported in endothelial cells, showing that 1 µM H_2_O_2_ could be beneficial while higher concentrations are harmful, and a similar biphasic effect of wavelengths was observed at identical fluence (10 J/cm^2^). These findings may be explained by an increased antioxidant production, by which the cells tolerate low and moderate but not high stress [10,43,44]. Our exciting results suggest that a low level of ROS is necessary to achieve the optimal vasodilation with 670 nm light. Since the complexes of the electron transport chain are considered major target chromophore molecules targeted by PBM, a mitochondrial redox state-dependent change in ATP accumulation is a typical primary effect of red/NIR exposure [4,5,6]. The ATP levels correlate with the fluence rather than with the wavelength. Using 636 nm and 825 lasers on primary dermal fibroblasts, the ATP concentrations remained steady up to 15 J/cm^2^, then decayed at higher, damaging fluences [10]. Here, we have not measured ATP changes as a response to 670 nm/6 J/cm^2^ (Figure 4b), indicating that the applied conditions were optimal to induce as little stress as just enough to stimulate vasodilation. The overall results also suggest that although the irradiation somewhat increased the mitochondrial ROS levels, this organelle is most likely not the central part of the red-light-regulated vasodilatory mechanisms; rather, cytosolic oxidations might be more relevant.

Cellular ROS sensor molecules can activate downstream transcription of oxidative stress protective genes (kinases, nuclear factors, etc.), raising the possibility that PMB can be pro-oxidant in the short term and antioxidant in long-term [42]. That would explain the temporal fading of red-light-dependent vasodilation we have experienced in vivo [15]. Future research directions will aim to map the downstream pathways. Our closest goal is to investigate the molecular background of red-light-assisted exocytosis. 

The main significance of this work is that it shows mechanistic evidence for the contribution of oxidative stress to the complex network of red-light-mediated vasodilation. Implementation of our findings will be the subject of further research on the efficiency of red-light therapy as a function of pre-existing oxidative stress using in vivo murine models on high glucose or high fat diets. 

One limitation of this study is the complexity of quantification of fluorescent microscopy data. Unfortunately, the lack of flow cytometry suitable antibodies for DMPO-coupled and SNO-Cys prevented us from using an alternative method. In the bigger picture, the main limitation of red-light treatment in vivo is the limited tissue penetration of the 670 nm light, which is 4–5 mm [45]. This depth allows the irradiation of the dermal vasculature, while a deeper reach requires the application of fiber optics-based catheters.

Since cardiovascular diseases remain a major cause for morbidity and mortality, mechanistic investigations to introduce new and efficient therapies are imperative. Cardiovascular co-morbid diseases, such as diabetes, atherosclerosis, etc., reduce proper endothelial function via impairment of the enzymatic production of critical vasodilatory species NO. Currently available pharmacological NO donors often suffer from limitations of possible drug tolerance or toxicity [46], while PBM offers utilization of alternative NO sources in a non-invasive way and without known side-effects. In addition, when surface irradiation is sufficient, a home-based application of light would provide a user-friendly treatment.

## 5. Conclusions

The application of 670 nm light triggers a mild oxidative stress with potential activation of subsequent signaling cascades in the endothelial cells which drive the formation, intracellular traffic, and release of vasodilatory-substance-containing exosomes. Based on these findings, a simple noninvasive therapy can be developed to treat subjects with circulation deficiency.

## Data Availability

The original contributions presented in the study are included in the article/Supplementary Materials, further inquiries can be directed to the corresponding author/s.

## Supplementary Materials

The following supporting information can be downloaded at: https://www.mdpi.com/article/10.3390/antiox13060668/s1, Figure S1: High glucose levels promote exosome formation, Figure S2: Comparison between the effects of mild (red light) and strong (H_2_O_2_) oxidative stress on early endosome membrane protein Rab5.
