# Supplementary material for: Vitamin E Attenuates Red-Light-Mediated Vasodilation: The Benefits of a Mild Oxidative Stress
Source: Antioxidants (Basel). 2024 May 29;13(6):668. doi: 10.3390/antiox13060668 (PMC11200653; doi:10.3390/antiox13060668)
Supplement: Supplementary file 1 [file antioxidants-13-00668-s001.zip › antioxidants-3001541-supplementary.pdf]

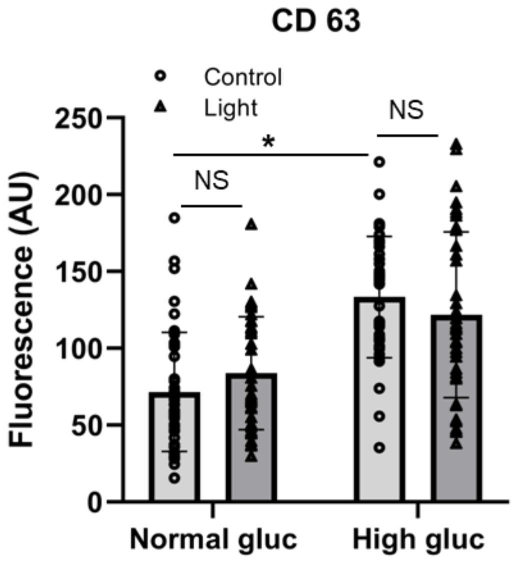

Figure S1. High glucose levels promote exosome formation. Endothelial cells (BAECs) were pre-incubated with D-glucose (15 mM, overnight) and exposed to light (670 nm, 6 J/cm^2). Expression of CD63 late exosome marker was immuno-labeled and detected with the fluorescence of secondary antibody. (*$p<0.05$ by using a one tailed unpaired Student's t test).

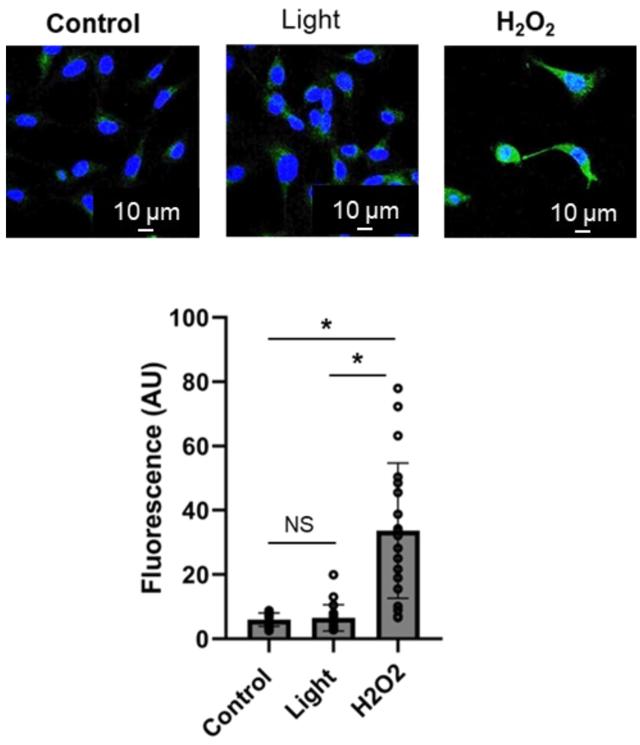

Figure S2. Comparison between the effects of mild (red light) and strong (H_2O_2) oxidative stress on early endosome membrane protein Rab5. Endothelial cells (BAECs) were exposed to light (670 nm, 6 J/cm^2), or treated with H_2O_2 (100 µM, 1 h). Rab5 levels were detected with immunofluorescence using the corresponding antibodies (green). Significantly higher intensities were recorded with vs. light exposure H_2O_2 (*$p<0.05$ by using a one tailed unpaired Student's t test). Nuclei were stained with DAPI (blue).
